# Preoperative Plasma Fibrinogen Level Represents an Independent Prognostic Factor in a Chinese Cohort of Patients with Upper Tract Urothelial Carcinoma

**DOI:** 10.1371/journal.pone.0150193

**Published:** 2016-03-01

**Authors:** Bo Zhang, Yi Song, Jie Jin, Li-Qun Zhou, Zhi-Song He, Cheng Shen, Qun He, Jun Li, Li-Bo Liu, Cong Wang, Xiao-Yu Chen, Yu Fan, Shuai Hu, Lei Zhang, Wei Yu, Wen-Ke Han

**Affiliations:** 1 Department of Urology, Peking University First Hospital, Beijing, People’s Republic of China; 2 Institute of Urology, Peking University, Beijing, People’s Republic of China; 3 National Urological Cancer Center, Beijing, People’s Republic of China; 4 Peking University Health Science Center, Beijing, People’s Republic of China; Sun Yat-sen University, CHINA

## Abstract

**Background:**

Increased plasma fibrinogen is thought to contribute to tumor progression and metastasis. The association of plasma fibrinogen with clinicopathological characteristics, and the optimal cutoff with an ideal predictive value has not been fully determined in patients with upper tract urothelial carcinoma (UTUC). We aimed to investigate the clinical significance of this parameter in a Chinese cohort of patients with UTUC.

**Methods:**

A retrospective study was conducted to analyze the clinical data of 184 operable UTUC patients in a Chinese cohort with a high incidence of chronic kidney disease (CKD). An optimal cutoff was set for further analysis according to validated web-based software. The associations of plasma fibrinogen with clinicopathological characteristics and survival were assessed. Multivariate analyses were performed to determine the independent prognostic factors.

**Results:**

Elevated plasma fibrinogen was significantly associated with tumor necrosis, lymph node involvement, and a higher preoperative CKD stage, pathological tumor stage and grade (all *P* < 0.05). Kaplan-Meier analysis showed that plasma fibrinogen ≥ 3.54 g/L predicted a poorer overall and cancer-specific survival than < 3.54 g/L (*P* < 0.001 for both). Multivariate analyses revealed that elevated preoperative plasma fibrinogen was an independent negative prognostic factor for overall survival (HR = 2.026; 95% CI: 1.226–3.349; *P* = 0.006) and cancer-specific survival (HR = 1.886; 95% CI: 1.019–3.490; *P* = 0.043).

**Conclusions:**

Increased plasma fibrinogen was an independent prognostic risk factor for poor outcomes in UTUC. This parameter may serve as an effective biomarker with easy accessibility for evaluating prognosis for patients with UTUC.

## Introduction

Upper tract urothelial carcinoma (UTUC) is a rare type of urothelial tumor. It accounts for only 5% of urinary tract urothelial carcinomas in western countries [1, 2]. Nevertheless, UTUC is relatively more common and is often complicated by chronic kidney disease (CKD) in the Chinese population [3–5]. This phenomenon is possibly attributed to widely used Chinese herbs that contain aristolochic acid [6, 7]. Despite non-metastatic UTUC patients not receiving a radical nephroureterectomy (RNU) with bladder cuff excision as a standard therapeutic approach, an adjuvant treatment might be indispensable for a high-risk subset. In addition to pathological T stage and tumor grade as established prognostic indicators of great importance, several other pathological parameters, such as tumor necrosis and lymphovascular invasion, were suggested to predict prognosis in UTUC [8–10]. Because of the impaired preoperative renal function and further damage after RNU, neo-adjuvant chemotherapy presented notable advantages in view of the limited opportunity to receive postoperative cisplatin-based chemotherapy [11]. Thus, this highlights the demand for discovering low cost, highly effective and easily accessible preoperative prognostic predictors.

Increasing evidence shows that there is an intimate connection between hemostatic factors and tumor biology. Fibrinogen, as a type of plasma glycoprotein, plays a vital part in clot formation, wound healing and supporting platelet aggregation. It is mainly produced by liver epithelium, and inflammatory stimuli could also promote fibrinogen synthesis in the lung and intestinal epithelium. Elevated plasma fibrinogen was reported to predict tumor progression, distant metastasis and poor oncological outcome in various malignancies [12–15]. Additionally, previous studies have shown plasma fibrinogen levels were related to certain pathological characteristics such as T stage, lymph node involvement, tumor grade, tumor size, and lymphovascular invasion in several cancers [16–18]. Two prior studies have also proven the prognostic value of plasma fibrinogen in UTUC in the Japanese and European populations [17, 18], whose carcinogenesis is quite different from the Chinese. Nevertheless, the potential connection between plasma fibrinogen level and inflammatory biomarkers has not yet been determined in UTUC.

We conducted a retrospective study to validate the prognostic impact of preoperative plasma fibrinogen level in a Chinese cohort of patients with UTUC and explore its potential association with clinicopathologic characteristics and systemic inflammation.

## Materials and Methods

### Patients and clinicopathologic evaluation

We retrospectively reviewed clinicopathologic and follow-up data of 211 patients with UTUC who received RNU from January 2006 to December 2008 in our center. To clarify, a pending related manuscript using the same cohort of patients was also submitted to PLOS ONE (PONE-D-15-26646). All patients underwent routine hematologic examination, ultrasound, cystoscopy, computer tomography/magnetic resonance imaging, and/or ureteroscopy with tissue biopsy before RNU. Patients with the following conditions were excluded from this analysis: distant metastasis at the initial diagnosis; no data on preoperative plasma fibrinogen levels; blood coagulation disorders; severe hypertension; autoimmune disease; liver disease; conservative surgery instead of RNU; neo-adjuvant chemotherapy. Finally, a total of 184 consecutive patients with pathologically diagnosed UTUC were enrolled in this study. Data regarding patients’ demographic and clinicopathologic characteristics, therapeutic regimens, and follow-up information were collected from a comprehensive database containing medical/pathological records of patients with UTUC. Glomerular filtration rate (GFR) was calculated using a CKD-EPI equation [19]. Preoperative CKD stages were classified on the basis of GFR (mL/min/1.73 m^2^) measured before RNU: stage 5 (GFR <15 or renal replacement therapy), stage 4 (15–29), stage 3 (30–59), stage 2 (60–89) and stage 1 (≥90). Tumors were staged according to the 2002 Union for International Cancer Control (UICC) TNM classification system. Tumor grading was assessed based on the World Health Organization (WHO) 1973 guidelines. This study was approved by the institutional ethics committee of Peking University First Hospital. As a retrospective analysis of routine data, a waiver of written informed consent was granted from the ethics committee. Patient records/information was anonymized and de-identified prior to analysis.

### Treatment and follow-up

All patients received open or laparoscopic RNU in this cohort. Based on a preoperative radiographic evaluation or intraoperative findings, a regional lymphadenectomy was performed in patients with enlarged lymph nodes. An extended lymphadenectomy was not routinely performed. Patients were assessed every 3 months for the first 2 years after the RNU, and annually thereafter. Evaluation items included routine blood tests, biochemical tests, urinalysis, cystoscopy, chest x-ray, and ultrasound/computed tomography/magnetic resonance imaging. The co-primary endpoints of this study were all-cause and cancer-specific deaths. Death causes were determined on the basis of death certificates or by patients’ treating clinicians. Overall survival (OS) and cancer-specific survival (CSS) periods were defined as the time from the date of RNU to the date of all-cause and cancer-specific deaths.

### Fibrinogen measurement

Plasma fibrinogen levels were routinely measured before surgery or diagnostic interventions as a part of a routine evaluation of coagulation function. For the plasma fibrinogen analysis, 3 mL of peripheral venous blood was collected on the day of admission to the hospital. Fibrinogen was measured based on the Clauss method as previously described [20]. The inter-assay coefficient of variation was <3% and <7.5% for the plasma control in the normal and pathological range, respectively.

### Statistical analysis

Continuous variables were reported as medians (IQR), and categorical variables were expressed as frequencies and percentages. The optimal cutoff value for plasma fibrinogen was determined using validated web-based software [21], which allowed us to treat it as a binary variable. In short, every possible cutoff point was examined for survival analysis, and the most significant (log-rank test) threshold was used throughout all further analyses. The associations of preoperative plasma fibrinogen levels with patients’ clinicopathological features were assessed by a nonparametric chi-squared test or Mann-Whitney U test. The relationships between plasma fibrinogen and inflammatory parameters (namely leukocyte, neutrophil and platelet counts, neutrophil-lymphocyte ratio and lymphocyte-monocyte ratio) were evaluated using a correlation analysis. OS and CSS were analyzed using the Kaplan-Meier method. A univariate analysis using the long rank test was performed to screen variables that may potentially predict prognosis. Then, those statistically significant variables were included in a multivariate Cox regression model to determine the independent prognostic risk factors. The optimal cutoff was determined using software based on R package, and the other statistical analyses were performed using the IBM Statistical Package for Social Sciences (SPSS) version 20.0. A result was considered statistically significant with *P* value of <0.05.

## Results

### Characteristics of the entire cohort

The demographic and clinicopathologic parameters of the 184 patients with UTUC are shown in Table 1. The complete study cohort consisted of 84 (45.7%) males and 100 (54.3%) females with a median age of 70 (61–75) years. The median preoperative plasma fibrinogen level of all cohorts was 3.52 (3.00–4.22) g/L. Ureter involvement was present in 85 (46.2%) patients, and multifocal lesions were noted in 46 (25.0%) patients. According to the 2002 UICC TNM classification system, 73 (39.7%) of patients had non-muscle-invasive disease (pTa-1), 111 (60.3%) had muscle-invasive disease (pT2-4), and 8 (4.3%) were diagnosed with lymph node metastasis. No distant metastases were observed at initial diagnosis in the cohort. According to the WHO 1973 grading guideline, 1 (0.6%), 116 (63.0%) and 67 (36.4%) patients were diagnosed with G1, G2 and G3 disease, respectively. In total, histological tumor necrosis was found in 30 (16.3%) patients.

**Table 1:** Demographic and clinicopathologic data of 184 patients with UTUC grouped by plasma fibrinogen level.

Variables	Plasma fibrinogen <3.54	Plasma fibrinogen≥3.54	*P* value
Number of patients	97	87	
Age, year	70 (60–74)	70 (64–75)	0.535
Follow-up, months	87 (69–99)	65 (28–83)	<0.001
Gender, female, n (%)	50 (51.5)	50 (57.5)	0.421
Body mass index, kg/m^2^	23.61 (22.04–26.45)	23.57 (21.26–26.56)	0.457
CHEH, n (%)	14 (14.4)	15 (17.2)	0.602
Preoperative CKD stage			0.002
No CKD/Stage 1/Stage 2	47 (48.5)	27 (31.1)	
Stage 3	40 (41.2)	37 (42.5)	
Stage 4/Stage 5	10 (10.3)	23 (26.4)	
Smoking history, n (%)	18 (18.6)	11 (12.6)	0.272
Previous or synchronous BUC, n (%)	16 (16.5)	13 (14.9)	0.773
ASA score ≥III, n (%)	21 (21.6)	25 (28.7)	0.268
Hydronephrosis, n (%)	34 (35.1)	45 (51.7)	0.023
Surgical procedure, open, n (%)	58 (59.8)	67 (77.0)	0.012
Ureter involvement, n (%)			0.044
Absent	59 (60.8)	40 (46.0)	
Present	38 (39.2)	47 (54.0)	
Multifocality, n (%)	22 (22.7)	24 (27.6)	0.443
Tumor architecture, n (%)			0.116
Papillary	87 (89.7)	71 (81.6)	
Sessile	10 (10.3)	16 (18.4)	
Pathological T stage, n (%)			0.010
Non-muscle-invasive (pTa-1)	47 (48.5)	26 (29.9)	
Muscle-invasive (pT2-4)	50 (51.5)	61 (70.1)	
Lymph node status, n (%)			0.028
N_0_/N_x_	96 (99.0)	80 (92.0)	
N_+_	1 (1.0)	7 (8.0)	
Tumor grade, n (%)			0.011
G1/G2	70 (72.2)	47 (54.0)	
G3	27 (27.8)	40 (46.0)	
Lymphovascular invasion, n (%)	12 (12.4)	16 (18.4)	0.256
Tumor necrosis, n (%)	10 (10.3)	20 (23.0)	0.020

ASA, American Society of Anesthesiologists; BUC, bladder urothelial carcinoma; CHEH, Chinese herbs exposure history; CKD, chronic kidney disease; UTUC, upper tract urothelial carcinoma.

### Association of preoperative plasma fibrinogen level with clinical characteristics

**Fig 1:**
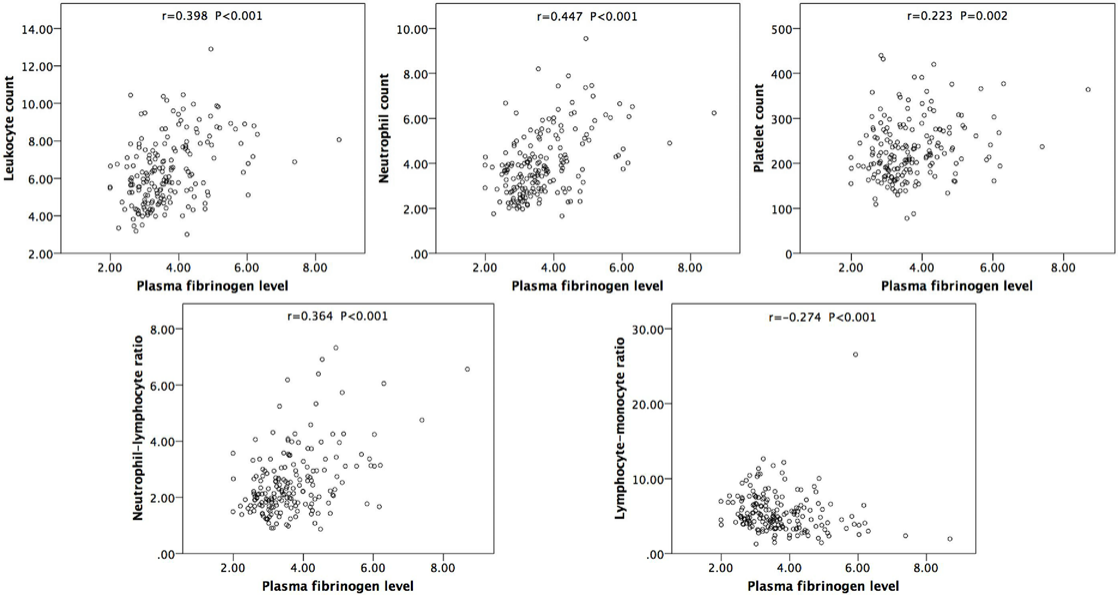
Correlation between plasma fibrinogen level and inflammatory parameters (i.e., leukocyte, neutrophil and platelet counts, neutrophil-lymphocyte ratio, and lymphocyte-monocyte ratio).

Based on the software mentioned above, the optimal cutoff value of 3.54 g/L was set, which best discriminated between survival and all-cause/cancer-specific death. Therefore, the cohort was divided into 2 groups. Of all the patients, 97 (52.7%) had a plasma fibrinogen level of less than 3.54 g/L, and 112 (67.1%) had a level of 3.54 g/L or above. There were no statistically significant differences in patient age, gender, smoking history, and lymphovascular invasion between the two groups (*P* > 0.05). Nevertheless, a high plasma fibrinogen level was significantly associated with tumor necrosis (*P* = 0.020), lymph node involvement (*P* = 0.028), and higher preoperative CKD stage (*P* = 0.002), pathological T stage (*P* = 0.010) and tumor grade (*P* = 0.011), which is shown in Table 1. Furthermore, Spearman’s correlation analysis showed that plasma fibrinogen level was positively correlated with neutrophil-lymphocyte ratio, leukocyte, neutrophil and platelet counts and was negatively correlated with lymphocyte-monocyte ratio (Fig 1).

### Association of preoperative plasma fibrinogen level with survival

**Fig 2:**
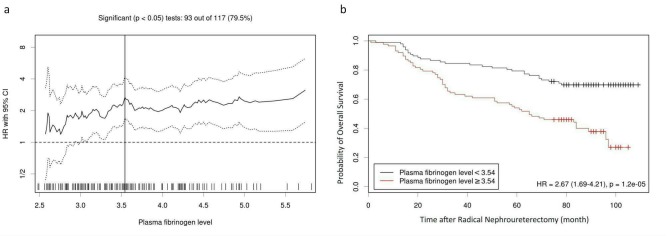
(a) Hazard ratio (HR) for OS based on each cutoff point of plasma fibrinogen level. A vertical line indicates the optimal cutoff value (3.54 g/L). (b) The Kaplan-Meier survival curve of OS stratified by the optimal cutoff value.

**Fig 3:**
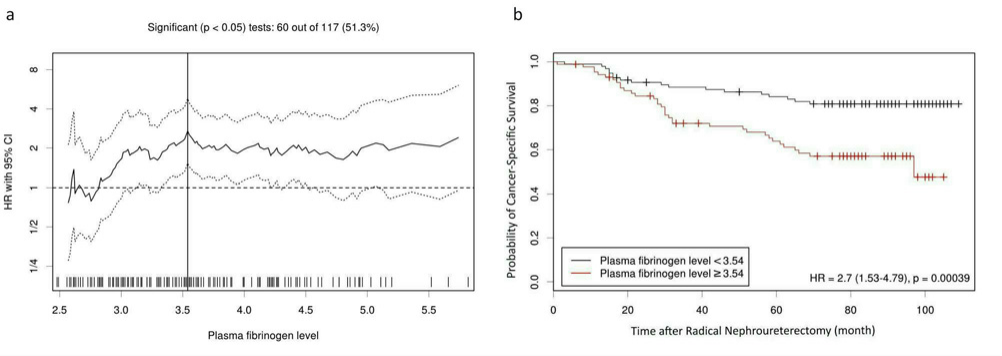
(a) Hazard ratio (HR) for CSS based on each cutoff point of plasma fibrinogen level. A vertical line indicates the optimal cutoff value (3.54 g/L). (b) The Kaplan-Meier survival curve of CSS stratified by the optimal cutoff value.

During the median follow-up of 78 (34–92) months, 82 (44.6%) died and 53 (28.8%) died of cancer-specific causes. In the entire cohort, the 5-year OS and CSS rate were 67.4±3.5% and 74.8±3.3%, respectively. The Kaplan-Meier survival analysis revealed that a plasma fibrinogen ≥ 3.54 g/L predicted significantly poorer OS and CSS than < 3.54 g/L (*P* < 0.001 for both), which is shown in Figs 2 and 3. In the patients with plasma fibrinogen < 3.54 and ≥ 3.54, the 5-year OS rates were 79.4±4.1% and 54.0±5.3%, and the 5-year CSS rates were 84.1±3.8% and 63.9±5.4%, respectively.

The univariate analysis identified a preoperative plasma fibrinogen level as a statistically significant predictor for both OS and CSS. To clarify the independent prognostic significance of preoperative plasma fibrinogen level for OS and CSS, multivariate analyses using a Cox proportional hazards regression model were performed to adjust for other prognostic indicators. The results of multivariate analyses revealed that preoperative plasma fibrinogen level was an independent risk factor in patients with UTUC. Patients with plasma fibrinogen ≥ 3.54 g/L had a higher risk of all-cause death (*P* = 0.006) and cancer-specific death (*P* = 0.043) than those with plasma fibrinogen < 3.54 g/L. Gender and pathological T stage were also independent prognostic indicators for OS and CSS. Older age (≥ 70) and preoperative CKD stage 4/5 were also identified as independent risk factors for OS. Tumorous ureter involvement was statistically significantly associated with poor CSS (Table 2).

**Table 2:** Cox proportional hazard univariate and multivariate analysis predicting OS and CSS in 184 patients with UTUC.

**OS**	**Univariate analysis**		**Multivariate analysis**		
	**χ^2^**	**P value**	**HR**	**(95%CI)**	***P* value**
Age, year (≥70 vs. <70)	7.149	0.008	2.023	1.259–3.250	0.004
Gender (male vs. female)	8.973	0.003	2.183	1.396–3.414	0.001
Preoperative CKD stage	10.632	0.005			
No CKD/Stage 1/Stage 2			(Reference)		
Stage 3			1.564	0.905–2.704	0.109
Stage 4/Stage 5			2.183	1.137–4.189	0.019
Cigarette smoking (yes vs. no)	0.683	0.409			
Previous or synchronous BUC (yes vs. no)	0.407	0.523			
ASA score (III vs. ≤II)	2.191	0.139			
Hydronephrosis (present vs. absent)	2.174	0.140			
Surgical procedure (laparoscopic vs. open)	1.526	0.217			
Ureter involvement (present vs. absent)	6.172	0.013	1.398	0.881–2.219	0.154
Multifocality (yes vs. no)	0.696	0.404			
Tumor architecture (papillary vs. sessile)	3.519	0.061			
T stage (T2-4 vs. Ta-1)	20.103	<0.001	2.718	1.486–4.972	0.001
Lymph node status (N_+_ vs. N_0_/N_x_)	3.562	0.059			
Tumor grade (G3 vs. G1/G2)	11.103	0.001	1.037	0.616–1.747	0.891
Lymphovascular invasion (present vs. absent)	0.003	0.958			
Tumor necrosis (present vs. absent)	3.407	0.065			
Plasma fibrinogen (≥3.54 vs. <3.54)	19.171	<0.001	2.026	1.226–3.349	0.006
**CSS**	**Univariate analysis**		**Multivariate analysis**		
	**χ**^**2**^	**P value**	**HR**	**(95%CI)**	***P* value**
Age, year (≥70 vs. <70)	2.727	0.099			
Gender (male vs. female)	15.372	<0.001	3.013	1.675–5.419	<0.001
Preoperative CKD stage	4.333	0.115			
No CKD/Stage 1/Stage 2					
Stage 3					
Stage 4/Stage 5					
Cigarette smoking (yes vs. no)	2.125	0.145			
Previous or synchronous BUC (yes vs. no)	0.047	0.828			
ASA score (III vs. ≤II)	1.503	0.220			
Hydronephrosis (present vs. absent)	6.519	0.011	0.956	0.507–1.800	0.888
Surgical procedure (laparoscopic vs. open)	1.086	0.297			
Ureter involvement (present vs. absent)	13.462	<0.001	2.556	1.324–4.935	0.005
Multifocality (yes vs. no)	0.768	0.381			
Tumor architecture (papillary vs. sessile)	6.788	0.009	1.383	0.651–2.940	0.399
T stage (T2-4 vs. Ta-1)	26.015	<0.001	4.369	1.755–10.879	0.002
Lymph node status (N_+_ vs. N_0_/N_x_)	9.142	0.002	2.335	0.881–6.193	0.088
Tumor grade (G3 vs. G1/G2)	12.117	<0.001	1.521	0.775–2.984	0.223
Lymphovascular invasion (present vs. absent)	0.029	0.864			
Tumor necrosis (present vs. absent)	3.513	0.061			
Plasma fibrinogen (≥3.54 vs. <3.54)	12.591	<0.001	1.886	1.019–3.490	0.043

ASA, American Society of Anesthesiologists; BUC, bladder urothelial carcinoma; CKD, chronic kidney disease; CSS, cancer-specific survival; OS, overall survival; UTUC, upper tract urothelial carcinoma.

## Discussion

Despite the developments in identifying biomarkers to predict survival, the conventional prognostic risk stratification heavily depends on postoperative parameters such as pathologic T stage, tumor grade, and N classification in patients with UTUC. However, more recommendations of neo-adjuvant chemotherapy due to decreased renal function after RNU highlights the significance of exploring effects and simple preoperative prognostic indicators in this cohort.

To the best of our knowledge, to date only two studies have revealed the prognostic value in UTUC in Japanese and Austrian populations [17, 18], whose pathogenesis is quite different from that in China. Before generally applying plasma fibrinogen for prognostic prediction, external validation in different ethnic subgroups is of great importance. Additionally, potential associations between plasma fibrinogen level and inflammatory biomarkers were not assessed in the two above-mentioned studies. Considering that the threshold of defining hyperfibrinogenemia does not necessarily best discriminate a poor and good oncological outcome, the optimal cutoff of plasma fibrinogen with an ideal predictive value remained to be determined in patients with UTUC.

Compared to two prior studies on the prognostic value of plasma fibrinogen in UTUC [17, 18], there were some differences in the cohort of this current study, which are illustrated in Table 3. In the cohort of our study, there were more female patients (54.3% vs. 26.6% and 39.5%), more tumors with localized pathological T stage (pTa-2: 78.3% vs. 46.8% and 61.3%) and lower tumor grades (G1-2: 63.6% vs. 39.6% and 54.4%) than in the Japanese and European cohorts. This was in accordance with the previous conclusion that Chinese UTUC patients are predominantly female with preferable pathological characteristics and relatively good oncological outcomes compared with those of western and Japanese populations. Similarly, our findings also revealed an association of high plasma fibrinogen with high pathological T stage and tumor grade. However, no statistically significant relationship between plasma fibrinogen and lymphovascular invasion was noted, which is inconsistent with what Tanaka et al. reported [18]. Importantly, we observed that elevated preoperative plasma fibrinogen was significantly associated with lymph node involvement and tumor necrosis. The relationship between increased plasma fibrinogen and lymph node metastasis was also observed in another study on gastric cancer [16].

**Table 3:** Comparison of this present study with those prior two on the prognostic value of plasma fibrinogen in UTUC.

Reference	Cohort	Plasma fibrinogen cutoff	Findings
Tanaka et al.[18]	218 Japanese UTUC patients (26.6% female) treated with RNU	4.50g/L was chosen after examining three cutoff values (3.90g/L, 4.20g/L and 4.50g/L, respectively).	Plasma fibrinogen ≥4.50 g/L was associated with ≥pT3 disease (HR = 3.56; 95% CI: 1.47–8.60; *P* = 0.005), positive lymphovascular invasion (HR = 2.99; 95% CI: 1.38–6.50; *P* = 0.005), tumor recurrence (HR = 2.00; 95% CI: 1.04–3.85; *P* = 0.038), and CSS (HR = 2.41; 95% CI: 1.20–4.85; *P* = 0.028) on multivariate analysis. Meanwhile, it was also associated with tumor length ≥ 30mm (*P* = 0.022), higher T stage (*P* = 0.007) and tumor grade (*P* < 0.001).
Pichler et al.[17]	167 European UTUC patients (39.5% female) treated with RNU or segmental ureteral resection	3.70g/L was set according to ROC curve.	Plasma fibrinogen ≥ 3.70 g/L was associated with CSS (HR = 3.00; 95% CI: 1.32–6.80, *P* = 0.008) and OS (HR = 2.48; 95% CI: 1.31–4.68; *P* = 0.005) on multivariate analysis. Meanwhile, it was also associated with higher T stage (*P* = 0.001) and tumor grade (*P* = 0.027).
This current study	184 Chinese UTUC patients (54.3% female) treated with RNU	3.54g/L was set according to a cutoff optimization tool [21].	Plasma fibrinogen ≥3.54 g/L was associated with CSS (HR = 1.886; 95% CI: 1.019–3.490, *P* = 0.043) and OS (HR = 2.026; 95% CI: 1.226–3.349; *P* = 0.006) on multivariate analysis. Meanwhile, it was also associated with lymph node involvement (*P* = 0.028), tumor necrosis (*P* = 0.020), and higher T stage (*P* = 0.010) and tumor grade (*P* = 0.011).

CSS, cancer-specific survival; OS, overall survival; UTUC, upper tract urothelial carcinoma.

Patients with malignancy suffered an abnormal state of hypercoagulability that contributes to tumor invasion, distant metastasis and other poor clinical outcomes [22, 23]. On the other hand, anticoagulants, including heparins and warfarin, have been reported to have antitumor and anti-metastatic effects [24]. Several possible mechanisms through which fibrinogen promotes tumor progression and leads to poor oncological outcome were raised. As an important molecular bridge, fibrinogen might facilitate stable adhesion between tumor cells, platelets and endothelial cells [25]. It has been reported that fibrinogen enhanced metastatic potential in part by impeding natural killer cell–mediated elimination of tumor cells [26]. It has been also suggested that fibrinogen and platelets facilitate each other in protecting tumor cells from natural killer cytotoxicity by forming thrombin, which depended on β3-integrins expressed on human cancer cells [27]. Endogenously synthesized fibrinogen interacts with several growth factors (including VEGF and FGF-2), which promotes cellular adhesion, proliferation and migration of tumor cells and neoplastic angiogenesis [28, 29]. In addition, plasma fibrinogen also plays a crucial role in the coagulation cascade, and elevated preoperative fibrinogen might also result in poor clinical outcome by inducing thromboembolism. It has been reported that high preoperative fibrinogen levels were related to elevated risk of pulmonary embolism, leading to a shortened overall survival [30].

In the Chinese population, patients with UTUC often also have CKD, which complicates the case and leads to a state of chronic systemic inflammation [3]. Growing evidence has proved the adverse impact of systemic inflammation on cancers [31, 32], including UTUC [33]. Importantly, genetic and pharmacologic studies have clarified the vital significance of fibrinogen in determining the extent of systemic inflammation, and its proinflammatory role has been reported in several types of malignancies [34]. Furthermore, certain inflammatory proteins, such as interleukin-6 or C-reactive protein, promote the production of fibrinogen. Our findings showed that plasma fibrinogen level is greatly associated with several inflammatory parameters, namely leukocyte count, neutrophil count, platelets count, NLR and LMR. Therefore, in addition to its direct adverse influence on oncological outcome, plasma fibrinogen reflects the degree of systemic inflammation that contributes to poor survival in patients with UTUC.

This current study has limitations: First, it was a single-center, small-sample and retrospective research, making certain unknown bias inevitable. Secondly, levels of some specific inflammatory proteins, such as interleukin-6 or C-reactive protein, were unavailable. Therefore, their association with plasma fibrinogen level was not evaluated in our study. Moreover, detailed information about steroid use that might affect plasma fibrinogen levels might be insufficient. However, even considering these limitations, our findings clearly suggest that an elevated plasma fibrinogen level is an independent risk factor for poor OS and CSS. We validated its prognostic value in a Chinese cohort of patients with UTUC. We also elucidated the association of fibrinogen with several inflammatory parameters, and underlined its significance in systemic inflammation in UTUC for the first time. This parameter should be applied to improve prognostic risk stratification models and assist clinicians in therapeutic strategy decision-making.

In conclusion, elevated preoperative plasma fibrinogen level is an independent risk factor of OS and CSS in patients with UTUC. As a low-cost and effective biomarker with easy accessibility, it should be monitored and applied in prognostic risk stratification methods in the future, which will assist clinician in therapeutic decision-making and improve treatment outcomes.
